# Fear of memories: the nature of panic in posttraumatic stress disorder

**DOI:** 10.3402/ejpt.v3i0.19084

**Published:** 2012-11-01

**Authors:** Amy Joscelyne, Siobhan McLean, Juliette Drobny, Richard A. Bryant

**Affiliations:** 1 School of Psychology, University of New South Wales, Sydney, Australia; 2 Department of Medical Psychology, Westmead Hospital, Sydney, Australia

**Keywords:** Panic attack, posttraumatic stress disorder, panic disorder, trauma memories

## Abstract

**Background:**

Although there is increasing evidence that panic attacks are common in posttraumatic stress disorder (PTSD), little is known if posttraumatic panic is comparable to panic attacks observed in panic disorder (PD).

**Objective:**

This study examined the cognitive responses to panic attacks in participants with PD and PTSD.

**Method:**

Participants with PD (*n*=22) and PTSD (*n*=18) were assessed on the Anxiety Disorder Interview Schedule for DSM-IV and subsequently administered the Agoraphobic Cognitions Questionnaire and a measure of fears related to trauma memories.

**Results:**

Although participants did not differ in terms of catastrophic appraisals about somatic sensations, PTSD participants were more likely to experience fears about trauma memories and being harmed by trauma again during their panic attacks than PD participants.

**Conclusions:**

These findings suggest that although PTSD participants fear somatic outcomes during panic attacks, their panic attacks are distinguished by a marked fear of trauma memories.

There is increasing evidence that panic attacks are common in people with posttraumatic stress disorder (PTSD). An analysis of the US National Comorbidity Survey found that 35% of people with PTSD had panic attacks in the past year, and this was linked to greater disability and comorbidity (Cougle, Feldner, Keough, Hawkins & Fitch, 2010). Many trauma survivors report experiencing panic attacks during the traumatic event; 90% of rape victims (Resnick, Falsetti, Kilpatrick & Foy, 1994) and 53% of motor vehicle and non-sexual assault survivors (Bryant & Panasetis, 2001) report at least four panic symptoms during the trauma. People with acute stress disorder (ASD) are more likely to experience panic attacks at the time of the trauma than those without ASD (Bryant & Panasetis, 2001). Furthermore, people with ASD also report more posttraumatic panic when compared to trauma survivors without ASD (Nixon & Bryant, 2003). Falsetti and Resnick (1997) found that 69% of treatment-seeking trauma survivors had experienced at least one panic attack in the 2 weeks prior to presenting for treatment. The importance of panic in the trajectory of PTSD responses is also highlighted by findings that initial dissociation mediates the relationship between peritraumatic panic and subsequent PTSD (Bryant et al., 2011), and peritraumatic panic predicts long-term mental health outcomes (Boscarino & Adams, 2009). Despite the relevance of panic attacks in PTSD, little is known about the nature of posttraumatic panic.

The intersection between PTSD and panic disorder (PD) is highlighted in recent years by fear circuitry models, which posit common etiologies and mechanisms across fear-based anxiety conditions, including PTSD and PD (Andrews, 2009). It is proposed that fear circuitry disorders share fear-conditioning processes at their point of origin such that otherwise benign stimuli are paired with an aversive experience; subsequent exposure to the conditioned stimuli signals threat and results in anxiety (Lanius, Frewen, Vermetten & Yehuda, 2010; Milad, Rauch, Pitman & Quirk, 2006). In the context of PTSD, this would involve reminders of the threat, whereas in the context of PD, it would require reminders of physical fears, such as choking, having a heart attack, or dying. This accords with models that posit that the arousal and panic experienced at the time of a traumatic experience become part of the conditioned stimuli, and thereafter somatic cues can trigger re-experiencing symptoms (Hinton, Hofmann, Pitman, Pollack & Barlow, 2008). Consistent with animal and human fear-conditioning research (Rauch & Drevets, 2009), fear circuitry disorders are characterized by excessive amygdala reactivity and impaired regulation of that response by the medial prefrontal cortex (Shin & Liberzon, 2010).

The major cognitive model of PD postulates that people catastrophically misinterpret somatic sensations to the extent that they fear that benign sensations are perceived as signals of impending death or severe illness (Clark, 1986, 1996). For example, sensations such as mild chest pain and dizziness may be viewed as being indicative of an impending heart attack. Supporting this model is the evidence that PD patients are more likely to interpret situations containing ambiguous internal stimuli as threatening (Clark et al., 1988; McNally & Foa, 1987; for a review, see McNally, 1994). Models of posttraumatic panic posit that panic that occurs at the time of trauma contributes to strong fear conditioning, and the somatic cues associated with the panic become associated with many other cues related to the traumatic experience (Falsetti, Resnick, Dansky, Lydiard & Kilpatrick, 1995; Jones & Barlow, 1990). These models propose that subsequent internal (e.g., emotions, physiological arousal, and cognitions) and external (e.g., places, objects, and smells) triggers elicit subsequent panic attacks, which in turn trigger trauma-related associations. This proposal is supported by evidence that 84% of a sample of trauma patients experiencing panic attacks reported that trauma reminders cued their panic attacks (Falsetti & Resnick, 1997).

Cognitive models are capable of explaining both PTSD and PD. The emphasis on cognitive responses in models of PD converges with cognitive PTSD models, which also propose that traumatic experiences can lead to catastrophic interpretations about the experience, the potential of future harm, and how one manages the effects of the traumatic experience (Ehlers & Clark, 2000). This is supported by evidence that maladaptive appraisals after trauma are predictive of subsequent PTSD (Dunmore, Clark & Ehlers, 1999; Ehlers, Mayou & Bryant, 1998; Warda & Bryant, 1998). Fear network models posit that mental representations of the feared content are highly connected and readily activated by cues that are related to the feared event; when activated, these representations can involve catastrophic appraisals about the feared event, thereby exacerbating the fear (Foa & Kozak, 1986). These representations can apply to traumatic or somatic representations, thereby being able to explain the cognitive responses of both PTSD and PD. It has been suggested that PTSD is characterized by a more widely activated fear network than other anxiety disorders as a result of the severity of the threat (Foa, Steketee & Rothbaum, 1989). Consistent with this proposal, trauma survivors display catastrophic appraisals about traumatic, somatic, and social events (Smith & Bryant, 2000).

An outstanding issue concerns the cognitive responses to posttraumatic panic attacks. Although PD models posit that the major mechanism underpinning the disorder is fear of aversive consequences of somatic events, it is possible that different fears underpin panic attacks in PTSD. Specifically, PTSD models emphasize that panic attacks cue conditioned responses that developed at the time of the traumatic experience and these attacks should accordingly trigger trauma memories. This hypothesis has indirect support from evidence indicating that inducing arousal in trauma survivors elicits trauma memories, as well as more flashback phenomena, in trauma victims with PTSD or ASD (Bremmer et al., 1997; Nixon & Bryant, 2005). On the basis of this hypothesis, we predicted that whereas panic attacks in the context of PD would be predominantly associated with fear of aversive outcomes from somatic perceptions, we expected that panic attacks in the context of PTSD would be associated with fear of trauma memories.

## Method

### Participants

The PD sample comprised 22 consecutively assessed participants (9 male and 13 female) of mean age 39.55 years (SD=13.08), who were seeking treatment at the Anxiety Treatment and Research Unit at Cumberland Hospital, Sydney; 21 participants met criteria for PD with agoraphobia and 1 had PD without agoraphobia. The PTSD sample comprised 18 consecutively assessed participants (8 male and 10 female) of mean age 42.22 years (SD=10.24), who were seeking treatment at the Traumatic Stress Clinic at Westmead Hospital, Sydney. Participants presented after motor vehicle accidents (*n*=11) or non-sexual assault (*n*=7). Inclusion criteria were (1) met criteria for PD or PTSD, (2) proficiency in English, (3) aged between 16 and 65 years, and (4) no diagnosis of organic mental disorder or psychosis. Patients in the PTSD group met DSM-IV diagnostic criteria for PTSD, did not meet criteria for PD, and had experienced at least one panic attack following their trauma. Patients in the PD group met DSM-IV criteria for either PD, with or without agoraphobia, but failed to meet criteria for a PTSD diagnosis. In terms of comorbidity of the PTSD participants, six participants had major depressive disorder and four had substance abuse. In terms of comorbidity of the PD participants, six participants were diagnosed with generalized anxiety disorder, five with major depressive disorder, two with obsessive–compulsive disorder, and one with social phobia. Four PD participants also reported past trauma, with two experiencing childhood abuse and two involved in a motor vehicle accident, but none reported PTSD symptoms related to these events.

### Measures

Diagnosis of PD and PTSD was ascertained using the Anxiety Disorder Interview Schedule for DSM-IV (ADIS-IV; Brown, Di Nardo & Barlow, 1994), which is a clinician-administered structured diagnostic interview following DSM-IV criteria. This schedule was also used to determine the presence of any comorbid anxiety disorders. The test–retest reliability of the ADIS-R (the predecessor of the ADIS-IV) ranges from 0.57 to 0.82 (di Nardo, Moras, Barlow, Rapee & Brown, 1993). PTSD severity was measured using the Clinician-Administered PTSD Scale (CAPS; Blake et al., 1995). The CAPS is a structured clinical interview that indexes the 17 symptoms described by the DSM-IV PTSD criteria.

Participants in both groups were also administered two questionnaires to index their panic-related cognitions. The Agoraphobic Cognitions Questionnaire (ACQ; Chambless, Caputo, Bright & Gallagher, 1984) consists of 14 cognitive statements that represent cognitive misappraisals of somatic symptoms. To assess trauma-related panic cognitions, a 12-item Traumatic Panic Cognitions Scale (TPCS) was developed. Items for this measure were based on proposals from experienced PTSD clinicians concerning the common fears that PTSD patients report during their panic attacks. This questionnaire comprised items that index the extent to which respondents may worry about traumatic memories or traumatic events occurring during a panic attack, such as, “Memories of the past are hurting me”, “I am reliving a terrible event”, and “I will never escape my memories”. The TPCS showed strong internal consistency, with a Cronbach's-α of 0.94. Both these questionnaires specifically indexed cognitions that occur during a panic attack and each utilized a five-point Likert scale (1=*never*, 2=*hardly ever*, 3=*sometimes*, 4=*often*, *5*=*always*).

### Procedure

Following informed written consent, participants were administered the ADIS-IV by clinical psychologists. Two weeks after the clinical interview, participants were administered the ACQ and TPCS in a random order of presentation.

## Results

### Participant characteristics

An independent sample *t*-test indicated no difference between the PTSD and PD groups in terms of their age (*t*(38)=−0.708, *ns*). There was also no difference in time since the onset of panic attacks between the PTSD (M=68.50 months, SD=49.56) and PD (M=60.41 months, SD=56.56) groups (*t*(38)=0.33, *ns*).

### Somatic reactions

Participants in the PTSD group (M=10.05, SD=2.95) and the PD group (M=9.00, SD=1.14) did not differ in terms of their total number of ADIS-IV assessed somatic panic symptoms (*t*(38)=1.42, *ns*). Table 1 presents the percentage of participants in both groups who reported each somatic symptom. Chi-square analyses that adopted a Bonferroni-adjusted α of *p*<.005 indicated that PD participants were more significantly likely to report numbing/tingling than PTSD participants (χ^2^(*N*=40)=7.78, *p*<.005).

**Table 1:** Number of participants reporting each panic symptom

	Panic disorder group, %	PTSD group, %
Palpitations	95.50	100.00
Sweating	77.30	100.00
Trembling	72.70	94.40
Shortness of breath	95.50	61.10
Choking	68.20	61.10
Chest pain	72.70	55.60
Nausea	86.40	83.30
Dizziness or faintness	86.40	72.20
Derealization/depersonalization	77.30	55.60
Fear of losing control or going crazy	72.70	33.30
Fear of dying	68.20	55.60
Numbing or tingling	81.80	38.90
Chills or flushes	50.00	88.90

### Cognitive reactions

The mean rating of each ACQ item is presented in Table 2. The PD (M=36.34, SD=11.83) and PTSD (M=32.50, SD=8.38) groups did not differ in terms of total ACQ scores, *t*(38)=1.25, ns. Multiple comparisons that adopted a Bonferroni-adjusted α of *p*<.005 indicated that PD participants reported a fear of passing out [*t*(38)=3.14, *p*<.005] and a fear of going blind [*t*(38)=3.20, *p*<.005] more often than PTSD participants.

**Table 2:** Mean rating of ACQ scores

	Panic disorder group		PTSD group	
		
	M	SD	M	SD
I am going to throw up	2.36	1.17	2.11	1.02
I am going to pass out	3.09	1.11	2.06	0.94
I must have a brain tumor	2.05	1.13	1.89	1.32
I will have a heart attack	3.23	1.31	3.00	1.14
I will choke to death	2.36	1.56	2.51	0.92
I am going to act foolish	3.18	1.50	2.44	1.04
I am going blind	1.91	1.11	1.06	0.24
I will not be able to control myself	3.41	1.44	2.89	1.08
I will hurt someone	1.55	1.06	2.11	1.41
I am going to have a stroke	2.68	1.52	1.50	1.04
I am going crazy	2.82	1.50	2.94	1.47
I am going to scream	2.41	1.56	2.44	1.62
I am going to babble or talk funny	2.55	1.53	3.00	1.19
I will be paralyzed by fear	3.05	1.56	2.56	1.42
Total score	36.64	11.83	32.50	8.38

Table 3 presents the mean rating of TPCS items. The PTSD participants (M=40.72, SD=10.06) scored higher than PD participants (M=26.78, SD=14.49) on TPCS items (*t*(38)=3.45, *p*<.001). PTSD participants were significantly more likely than PD participants to report that during a panic attack they relived a terrible event [*t*(38)=−2.94, *p*<.005], that memories of their past were hurting them [*t*(38)==−3.91, *p*<.001], that something was going to hurt them again [*t*(38)=−3.55, *p*<.001], that their memories were driving them crazy [*t*(38)=−3.11, *p*<.005], that they feel trapped in the past [*t*(38)=−3.06, *p*<.005], and that they cannot block out terrible memories [*t*(38)=−3.03, *p*<.005].

**Table 3:** Mean traumatic panic cognitions scale scores

	Panic disorder group		PTSD group	
		
	M	SD	M	SD
I cannot cope with my past	2.14	1.39	3.11	1.32
I am reliving a terrible event	2.05	1.25	3.22	1.26
Memories of my past are hurting me	2.41	1.40	3.83	0.71
Something is going to hurt me again	2.23	1.51	3.67	0.91
I cannot cope with my memories	2.41	1.33	3.44	1.04
I am going back to a bad experience in my past	2.09	1.31	2.94	1.35
I cannot escape a distressing thing in my past	2.23	1.19	3.22	1.17
My memories are making me crazy	2.14	1.32	3.39	1.20
I am weak because I can't get over my past	2.27	1.49	3.50	1.29
I will never escape my memories	2.41	1.40	3.56	1.10
I am trapped in the past	2.09	1.27	3.33	1.28
I cannot block out terrible memories	2.32	1.36	3.50	1.04
Total Score	26.77	14.49	40.72	10.06

## Discussion

PD and PTSD participants reported comparable somatic symptoms during their panic attacks. This finding accords with previous evidence that PTSD and PD participants report comparable somatic experiences during panic attacks (Falsetti & Resnick, 1997). Despite this similarity in somatic response, PTSD participants reported marked fear of trauma memories more than PD participants. PTSD participants tended to report that during their panic attacks, they experience traumatic intrusions, fears that they would be harmed again, and an inability to control trauma memories. This pattern suggests that the cognitive profile of panic attacks in PTSD may be qualitatively different from the panic attacks typically observed in PD. Whereas PD is characterized by fear of aversive consequences of somatic events, PTSD participants predominantly fear the consequences of trauma memories. This observation is consistent with models of posttraumatic panic that posit that panic reactions at the time of trauma are conditioned with the traumatic event, and that subsequent panic reactions will trigger associated memories (Falsetti et al., 1995; Jones & Barlow, 1990).

Contrary to expectation, PTSD participants reported comparable catastrophic interpretations about somatic sensations as PD participants. This pattern suggests that panic attacks in PTSD participants also involve maladaptive cognitive responses about somatic events. Previous work has demonstrated that people with posttraumatic stress exaggerate the probability of aversive events occurring and the aversive consequences of these events (Warda & Bryant, 1998). Importantly, this pattern extends to exaggerating aversive outcomes from somatic events, as well as physical harm (Smith & Bryant, 2000). This pattern is consistent with evidence that people with ASD have higher anxiety sensitivity scores than those without ASD (Bryant & Panasetis, 2001), and that anxiety sensitivity scores are strongly predictive of posttraumatic panic (Nixon & Bryant, 2003). Taken together, these findings suggest that the fear conditioning that occurs during the traumatic event encompasses the aversive outcomes of somatic events, and in this sense the panic attacks in PTSD participants share the fear of somatic catastrophes that are observed in PD panic attacks. The panic attacks in PTSD participants, however, are additionally characterized by the fear of trauma memories.

These findings may have implications for clinical management of posttraumatic panic. PD is traditionally treated using interoceptive exposure that allows the patient to learn that the somatic sensations do not result in the feared outcome (Barlow & Craske, 1988). More recent treatment protocols have been developed to specifically treat comorbid PTSD and PD, which have commenced with interoceptive exposure prior to treating traumatic stress by trauma-focused exposure (Falsetti, Resnick, Davis & Gallagher, 2001). This approach presumes that the panic should be managed via panic management strategies prior to addressing the trauma symptoms. In contrast to this approach, the current findings suggest that effective management of panic in PTSD may occur after imaginal exposure that involves prolonged exposure to the trauma memories, and may not directly require interoceptive exposure. It is worth noting that whereas prolonged exposure is efficacious in treating PTSD symptoms (Harvey, Bryant & Tarrier, 2003), treatment studies to date have not indexed the influence of trauma-focused exposure therapy on posttraumatic panic attacks. Interestingly, recent treatment studies of PD have found that panic-focused cognitive behavior therapy is beneficial in reducing comorbid anxiety conditions; however, these studies have not included PTSD patients (Craske et al., 2007; Tsao, Mystkowski, Zucker & Craske, 2005). We note that whereas our PTSD participants suffered panic attacks, they did not meet the criteria for PD; patients with comorbid PTSD and PD may require interventions that do involve interoceptive exposure because of the significant ongoing fear of somatic outcomes. The extent to which prolonged exposure of trauma memories will reduce posttraumatic panic attacks, and PD, remains to be tested by randomized controlled trials.

This study's conclusions are qualified by the small sample size; future studies that employ larger samples may provide a more robust test of differences in cognitions, and also allow analysis of cognitions about panic following different types of trauma. We note that we relied on a measure of posttraumatic panic cognitions that has not been validated. Development of a measure of these cognitions that is subsequently validated and used in samples of PTSD and PD patients would strengthen the current findings. These limitations notwithstanding, the current findings suggest that posttraumatic panic needs to be understood as a different experience from panic attacks in PD. The fear of trauma memories experienced during posttraumatic panic suggests that these memories may need to be the focus of therapy intervention. The extent to which prolonged exposure of trauma memories resolves posttraumatic panic remains to be tested.
